# The Musk-Deer

**Published:** 1856-08

**Authors:** Fred. Markham

**Affiliations:** 32d Regiment


### SELECTIONS.

[From the Western Lancet.]

*The Musk-Deer.*

This little persecuted animal would probably have been left undisturbed to pass a life of peace and quietness in its native forests, but for the celebrated perfume with which nature has provided it. Its skin being worthless from its small size, the flesh alone would hold out no inducement for the villagers to hunt it while larger game was more easily procurable; and its comparative insignificance would alike have protected it from the pursuit of the European sportsman. As the musk, however, is the most valuable of all, no animal is so universally sought after in every place it is known to inhabit. Musk is in demand in nearly every part of the world, yet little, I believe, is known of the nature and habits of the animal that produces it.

The musk-deer is rather more than three feet long, and stands two high, at the shoulder; but they vary considerably in size, those found in thick, shady woods being invariably larger than those on rocky open ground. The head is small, the ears long and erect. The male has a tusk depending from each upper jaw, which, in a full-grown animal, is about three inches long, the thickness of a goose-quill; sharp-pointed, and curving slightly backwards. The general color is a dark speckled brownish-gray, deepening to nearly black on the hind-quarters, where it is edged down the inside of the thighs with reddish-yellow. The throat, belly and legs are of a lighter gray. Legs long and slender; toes long and pointed; the hind heels are long, and rest on the ground as well as the toes. The fur is composed of thick spiral hairs, not unlike miniature porcupine quills; they are very brittle, breaking with a slight pull, and so thickly set that numbers may be pulled out without altering the outward appearance of the fur. The fur is much longer and thicker on the hind parts than the fore, and gives the animal the appearance of being much larger in the hind-quarters than the shoulder. The tail, which is not seen unless the fur is parted, is an inch long, and about the thickness of a thumb; in females and young animals, it is covered with hair, but in adult males is quite naked, except a slight tuft at the end; and often covered, as well as all the parts near it, with a yellowish waxy substance.

The musk, which is much better known than the deer itself, is only found in adult males; the females have none, neither has any portion of their bodies the slightest odor of musk. The dung of the males smells nearly as strong as musk, but, singularly enough, neither in the contents of the stomach, nor bladder, nor in any part of the body, is there any perceptible scent of musk. The pod, which is placed near the navel, and between the flesh and the skin, is composed of several layers of thin skin, in which the musk is confined, and has much the appearance of the craw or stomach of a partridge, or other small gallinaceous bird, when full of food. There is an orifice outwards through the skin, into which, by a slight pressure, the little finger will pass, but it has no connection whatever with the body. It is probable that musk is at times discharged through this orifice, as the pod is often found not half full, and sometimes even nearly void. The musk itself is in grains, from the size of a small bullet to small shot, of irregular shape, but generally a dark reddish-brown color, but when taken out of the pod and kept for any length of time, becomes nearly black. In autumn and winter the grains are firm, hard, and nearly dry, but in summer they become damp and soft, probably from the green food the animals then eat. It is formed with the animal, as the pod of a young one, taken out of the womb, is plainly distinguishable, and indeed is much larger in proportion than in grown-up animals. For two years the contents of the pod remain a soft, milky substance, with a disagreeable smell. When it first becomes musk, there is not much more than the eighth of an ounce; as the animal grows, it increases in quantity; and in some individuals as much as two ounces are found. An ounce may be considered as the average from a full-grown animal; but as many of the deer are killed young, the pods in the market do not perhaps contain, on an average, more than half an ounce. Though not so strong, the musk of young animals has a much pleasanter smell than that of old ones; but difference of food, climate, or situation, as far as my experience goes, does not at all affect the quality.

From the first high ridge above the plains, to the limits of forests on the snowy range, and for perhaps the whole length of the chain of the Himalayas, the musk-deer may be found upon every hill of an elevation above 8,000 feet, which is clothed with forest. On the lower ranges it is comparatively a rare animal, being confined to near the summits of the highest hills, as we approach the colder forests near the snow; but it is nowhere particularly numerous; and its retired and solitary habits make it appear still more rare than it really is. Exclusively a forest animal, it inhabits all kinds of forest indiscriminately, from the oaks of the lower hills to the stunted bushes near the limits of vegetation. If we may judge from their numbers, the preference seems to be given to the birch forests, where the underwood consists chiefly of the white rhododendron and juniper.

In many respects, they are not unlike hares in habits and economy. Each individual selects some particular spot for its favorite retreat, about which it remains still and at rest throughout the day, leaving it in the evening to search for food, or to wander about, returning soon after daylight. They will occasionally rest for the day in any place where they may happen to be in the morning, but in general they return to near the same spot almost every day, making forms in different quarters of their retreat a little distance from each other, and visiting them in turn. Sometimes they will lie under the same tree or bush for weeks together. They make forms in the same manner as hares, levelling with their feet a spot large enough for the purpose, if the ground is too sloping. They seldom, if ever, lie in the sun, even in the coldest weather, and their forms are always made where there is something to shelter them from its rays. Towards evening they begin to move, and during the night appear to wander about a good deal, from top to bottom of the hill, or from one side to another. The Puharries believe that they come to such places to play and dance with each other, and often set their snares along the edge of such a ledge or precipice, in preference to the forest.

If not walking leisurely and slowly along, the musk-deer always goes in bounds, all fours, leaving and alighting on the ground together. When at full speed, these bounds are sometimes astonishing, for so small an animal. On a gentle slope I have seen them clear a space of more than sixty feet at a single bound, for several successive leaps, and spring over bushes of considerable hight at the same time. They are very sure footed, and although a forest animal, in traveling over rocky and precipitous ground, have perhaps no equal. Where even the burrell is obliged to move slowly and carefully, the musk-deer bounds quickly and fearlessly; and although I have often driven them on to rocks which I thought it impossible they could cross, they have invariably found a way in some direction, and I never knew an instance of one missing its footing, or falling, unless wounded.

They eat but little compared to other ruminating animals, at least one would imagine so, from the small quantity found in their stomachs, the contents of which are always in such a pulpy state, that it is impossible to tell what food they prefer. I have often shot them whilst feeding, and found in the mouth or throat various kinds of shrubs and grasses, and often the long white moss that hangs so luxuriantly from the trees in the higher forests. Roots also seem to form a portion of their food, as they scratch holes in the ground, like many of the hill pheasants. The Puharries believe that the males kill and eat snakes, and feed upon the leaves of the "kedar patta," a small and very fragrant smelling laurel, and that the musk is produced by this food. They may probably eat the leaf of this laurel, amongst other shrubs; but from the few occasions upon which I have seen this laurel stripped of any portion of its leaves, it does not appear to afford a very favorite repast. Their killing snakes is, doubtless, quite fabulous.

The young are born either in June or July, and almost every female brings forth yearly, and often twins. These are always deposited in separate places some distance from each other, the dam herself keeping apart from both, and only visiting to give them suck. Should a young one be caught, its bleating will sometimes bring the old one to the spot, but I never knew an instance of one being seen abroad with its dam, or of two young ones being seen together. Their solitary habits are innate, for if a fawn is taken young and suckled by a sheep or goat, it will not for some time associate with its foster-dam, but as soon as satisfied with sucking, seeks some spot for concealment. It is amusing to see them suck; all the while they keep leaping up and crossing their fore legs rapidly over each other. They are rather difficult to rear, as many, soon after they are caught, go blind and die.

In most of the hill-states, the musk-deer is considered as royal property. In some, the Rajahs keep men purposely to hunt it; and in Gurwhal a fine is imposed upon any Puharrie who is known to have sold a musk pod to a stranger—the Rajah receiving them in lieu of rent.

In some districts they are hunted down with dogs, but snaring is by far the most common method practised for their capture. A few are occasionally shot by the village shikaries when in pursuit of other animals, but the matchlock is seldom taken out purposely to hunt musk-deer, for a hill shikarie does not carry the match lighted, and the deer being generally come upon face to face, almost every one would get away before he could strike a light and apply it to the match. In snaring, a fence about three feet high, composed of bushes and branches of trees, is made in the forest, generally along some ridges, and often upwards of a mile in length. Openings for the deer to pass through are left every ten or fifteen yards, and in each a strong hempen snare is placed, tied to a long stick, the thick end of which is firmly fixed in the ground, and the smaller, to which the snare is fastened, bent forwards to the opening, so that the deer, when passing through, treads upon some small sticks which hold it down, the catch is set free, the stick springs back and tightens the snare round the animal's leg. Besides the musk-deer, numbers of the forest pheasants, moonals, corklass, and argus are caught in these snares; they are visited every third or fourth day, and it is seldom that the owners return without something or other. The polecats often find out the snares, and after once tasting the feast, if not destroyed soon, become a terrible annoyance, tracing the fence almost daily from end to end, and seizing on every thing caught, they are often caught themselves, but immediately bite the snare in two, and escape. Musk-deer are frequently lost to the snarers in this manner, for when one is eaten by the polecats, the pod is torn to pieces, and the contents scattered on the ground. No animal swallows the musk, and when a deer has been killed and eaten by a leopard or other animal, if the ground be carefully examined, much of the musk may be picked up. Insects and maggots also leave it untouched. I once found what I thought was a newly killed musk-deer, but on examination, I discovered it was merely the skin and skeleton of one which, from its dry and withered state, must have been dead some months; the flesh had been completely eaten away by maggots, but the musk-pod was entire.

The musk-pods which reach the market through the hands of the native hunters are generally inclosed in a portion of the skin of the animal, with the hair or fur left on it. When they have killed a musk-deer, they cut round the pod, and skin the whole of the belly. The pod comes off attached to the skin, which is then laid with its fleshy side on a flat stone, previously heated in the fire, and thus dried without singing the hair. The skin shrinks up from the heat into a small compass, and is then tied or stitched round the pod, and hung up in a dry place until quite hard. This is the general method of preparing them, but some put the pod into hot oil instead of laying it on a hot stone, but either method must deteriorate the quality of the musk, as it gets either completely baked or fried. It is best both in appearance and smell, if the pod is at once cut from the skin, and allowed to dry of itself.

The musk received from the Puharries is greatly adulterated, and pods are often made altogether counterfeit; and as they are generally sold without being cut open, it is scarcely possible to detect the imposture at the time. I have often seen pods offered for sale, which were merely a piece of musk-deer skin filled with some substance, and tied up to resemble a musk.pod, with a little musk rubbed over to make it smell. These are easy to detect, from there being no navel on the skin, it being cut from any part of the body. But the musk is sometimes taken out of real pods, and its place supplied by some other substance, and these are difficult to detect, even if cut open, as whatever is put in is made to resemble musk in appearance, and a little genuine added makes it smell nearly as strong. Some have only a portion of the musk taken out, and its place thus supplied; and others have all the musk left in, but something added to increase the weight. Even in the hills where it is produced, so little do the generality of the people know of musk, that I have often seen the Puharries about Gangoutre sell to the pilgrims, to men from the lower hills, and even to their own neighbors, small portions of what they called musk, but what was merely some substance resembling it, with a little genuine musk scattered over it. Of this stuff they would sell about a quarter of a tolah for a rupee, or about twenty shillings an ounce.

The substances commonly used for adulteration or to fill the counterfeit pods are, blood boiled, or baked on the fire, then dried, beaten to a powder, kneaded into a paste, and made into grains and coarse powder to resemble genuine musk; a piece of the liver or spleen prepared in the same manner; dried gall, and a particular part of the bark of the apricot-tree, pounded and kneaded as above. The dried paste from which common oil has been extracted, called "peena," is also used, and lumps of this are often, without further preparation, thrust into a pod through the orifice in the skin, to increase the weight. Sometimes no care is taken to give the material employed in filling a counterfeit pod even the appearance of musk. A gentleman once showed me a pod he had bought from a Puharrie at Missouri; on my telling him it was counterfeit, he cut it open, and found it filled with hookah tobacco.—*Pharm. Journ.*, *from Shooting in the Himalayas: a Journal of Sporting Adventures and Travel in Chinese Tartary, Ludac, Thibet, Cashmere, etc* By Col. Fred. Markham, C. B., 32d Regiment.

